# Outcomes of medical patients requiring emergency intubation in a rural Irish hospital

**DOI:** 10.1186/cc14286

**Published:** 2015-03-16

**Authors:** A O'Connor, MP D'Alton, B Carey

**Affiliations:** 1 Bantry General Hospital, Co. Cork, Ireland

## Introduction

Bantry General Hospital (BGH) is a small rural hospital serving a large, geographically isolated part of southwest Ireland. Following an influential national review of adult critical care services [1], a protocol was introduced in late 2010 mandating the immediate transfer of all medical patients intubated on an emergency basis to a large critical care centre 100 km away. Similar mandatory transfer protocols were introduced at the same time throughout the island of Ireland but few data are available regarding patient outcomes. We designed a study to look at the outcomes of all patients encompassed by the protocol at BGH.

## Methods

We retrospectively reviewed the charts and electronic data of medical patients requiring emergency intubation at BGH from November 2010 to December 2013. We recorded the following data: age, sex, admission diagnosis, comorbidities, time delay to transfer, in-transit mortality, length of stay, survival to discharge and 1-month and 6-month mortality.

## Results

Forty-five patients (31 male) were included with a mean age of 67 ± 15 years. The commonest admission diagnoses were sepsis (10), cardiogenic shock (10), primary respiratory failure (nine) and intracranial haemorrhage (eight). The median transfer delay time was 47 minutes. Only 27 (60%) patients were actually transferred and they were significantly younger than nontransferred patients (62 vs. 73 years, *P *= 0.02). In-transit mortality was zero. Mean length of stay in the critical care centre was 14.8 ± 16.8 days. Survival to discharge was significantly higher in transferred (14/27) compared with nontransferred (3/18) patients (52% vs. 17%, *P *= 0.017). Overall mortality rates were 62% and 69% at 1 and 6 months respectively and were significantly lower in the transferred group (*P *= 0.02).

## Conclusion

Overall mortality rates of medical patients intubated urgently at BGH were high. Forty per cent of intubated patients were not transferred, indicating significant modification of the protocol over time. Patients transferred to the critical care centre were younger and had significantly better outcomes than patients remaining in BGH, probably due to decisions not to transfer patients with poor prognoses. Most patients who survived to discharge were still alive 6 months later.
